# Hand-assisted laparoscopic resection versus total laparoscopic gastric surgery for primary gastric gastrointestinal stromal tumors (GISTs): an analysis from a high-volume institution

**DOI:** 10.1186/s12893-022-01668-y

**Published:** 2022-06-07

**Authors:** Ya-Jun Zhao, Yong-Qiang Qiu, Li-Ying Zhong, Wen-Ze Zheng, Lv-Ping Zhuang, Zhong Wei, Zhong-Liang Ning

**Affiliations:** 1 Department of Gastrointestinal Surgery, The First Affiliated Hospital of University of Science and Technology of China, Division of Life Sciences and Medicine, University of Science and Technology of China, Hefei, 230031 Anhui China; 2 Department of Anesthesiology, The First Affiliated Hospital of Fujian Medical University, Fuzhou, China; 3 Department of Clinical Medicine, Xiamen Medical College, Xiamen, China; 4 The Graduate School of Fujian Medical University, Fujian Medical University, Fuzhou, China; 5 Department of Neurology, Fujian Medical University Union Hospital, Fuzhou, Fujian China

**Keywords:** Gastric GIST, Hand-assisted laparoscopic surgery, Total laparoscopic surgery, Surgical outcome

## Abstract

**Background:**

Laparoscopic resection of gastric gastrointestinal stromal tumors (GISTs) is technically feasible and associated with favorable outcomes. We compared the clinical efficacy of hand-assisted laparoscopic surgery (HLS) and total laparoscopic surgery (TLS) for gastric GISTs.

**Methods:**

We retrospectively analyzed the clinical data of 69 consecutive patients diagnosed with a gastric GIST in a tertiary referral teaching hospital from December 2016 to December 2020. Surgical outcomes were compared between two groups.

**Results:**

Fifty-three patients (TLS group: n = 36; HLS group: n = 17) were included. The mean age was 56.9 and 58.1 years in the TLS and HLS groups, respectively. The maximum tumor margin was significantly shorter in the HLS group than in the TLS group (2.3 ± 0.9. vs. 3.0 ± 0.8 cm; *P* = 0.004). The operative time of the HLS group was significantly shorter than that of the TLS group (70.6 ± 19.1 min vs. 134.4 ± 53.7 min; *P* < 0.001). The HLS group had less intraoperative blood loss, a shorter time to first flatus, and a shorter time to fluid diet than the TLS group (*P* < 0.05). No significant difference was found between the groups in the incidence or severity of complications within 30 days after surgery. Recurrence or metastasis occurred in four cases (HLS group; n = 1; TLS group; n = 3).

**Conclusions:**

This study demonstrated that compared with TLS, HLS for gastric GISTs has the advantages of simpler operation, shorter operative time, and faster postoperative recovery.

## Introduction

Gastrointestinal stromal tumors (GISTs) can arise anywhere along the gastrointestinal (GI) tract, and gastric GISTs (60%) are the most common type [1]. Current surgical methods to treat gastric GISTs include traditional open surgery, hand-assisted laparoscopic surgery (HLS), and total laparoscopic surgery (TLS). TLS has gradually replaced open surgery due to its advantages of less intraoperative blood loss and faster postoperative recovery [2–4]. A gastric wedge resection is a common approach for laparoscopic gastric GIST treatment and involves removing the tumor and the surrounding gastric tissue together, which can preserve the function of the stomach to the greatest extent. However, in the process of performing a laparoscopic gastric wedge resection, surgeons, especially newly trained surgeons, are unable to directly palpate the tumor, resulting in incorrect judgments of tumor size and scope. This may result in the excessive removal of normal stomach tissue. For gastric GISTs in specific areas such as the fundus, cardia, antrum, and pylorus, a total gastrectomy or subtotal gastrectomy together with digest tract reconstruction is required. This increases surgical trauma and costs, while also affecting the postoperative quality of life. Hand-assisted laparoscopic technology was previously applied to radical GI tumor surgery, which is a transition between open surgery and TLS. Recently, with the advancement of laparoscopic technology, the use of HLS has gradually decreased in the field of radical resection of intestinal malignancies [5].

Since HLS has been used to treat gastric GISTs, it has been found that it not only has the advantages of minimally invasive surgery, but it also allows surgeons to make accurate judgments about the scope and size of the tumor during the operation, which protects normal stomach tissue and prevents intraoperative tumor rupture to a certain extent [5]. Therefore, this study was conducted to compare the clinical efficacy of hand-assisted and total laparoscopic gastric wedge resection for gastric GISTs in a high-volume medical center.

## Patients and methods

### Population and covariates

**Fig. 1:**
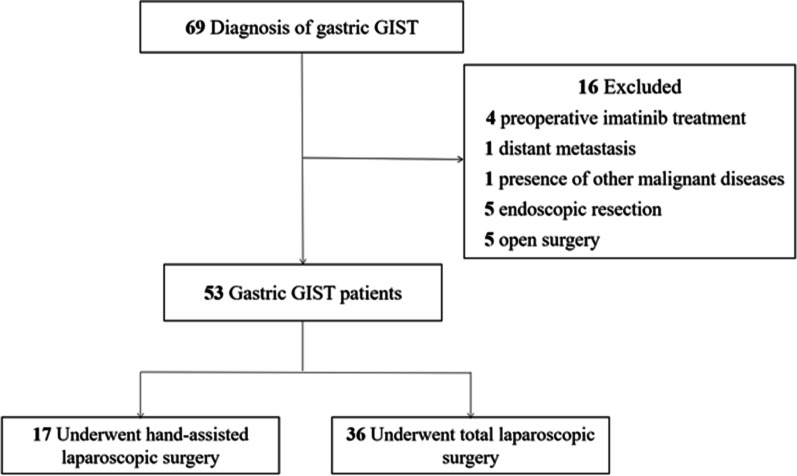
Study flowchart

Between December 2016 and December 2020, 69 consecutive patients with primary gastric GISTs were treated at the First Affiliated Hospital of the University of Science and Technology of China. The following exclusion criteria were applied: (1) received preoperative imatinib treatment, (2) presence of distant metastasis or another malignant disease, (3) received endoscopic resection, and (4) underwent open surgery. Finally, 53 patients with primary gastric GISTs who underwent laparoscopic surgery were included in the analysis. The patients were divided into the HLS group (n = 17 patients) and the TLS group (n = 36 patients) according to the surgical method used. The selection scheme is shown in Fig. 1. This study was approved by the Institutional Review Board of the First Affiliated Hospital of the University of Science and Technology of China (IRB number: 2021-WCK-01).

All patients underwent routine preoperative examinations, including an upper GI endoscopy and an upper GI angiography to assess tumor location. Simultaneously, an ultrasound gastroscopy was performed to determine the size and depth of the invasion of the tumor. For patients whose tumor location or margin was unclear before surgery, we would identify the location or measurement intraoperatively with the help of an intraoperative gastroscopy. The indications for HLS surgery in this study included patients with various gastric stromal tumors, especially those expected to have adhesion and invasion of the tumor and surrounding tissues, or large tumors. The indication for TLS surgery included stromal tumors with tumor diameter ≤ 5 cm or tumor ≥ 5 cm but located in the anterior wall of the gastric body and the greater curvature of the easy-to-operate site. All patients were informed of the relevant surgical risks and costs, and each patient had the right to choose the type of surgery: TLS or HLS. All patients provided informed consent before surgery. The postoperative patients were managed using the same clinical pathway. All patients enrolled in this study were operated on by the chief surgeon (Zhong-Liang Ning), with an experience of more than 50 total and 30 hand-assisted laparoscopic gastrectomies before the study, having been gone through the learning curve [6, 7].

**Fig. 2:**
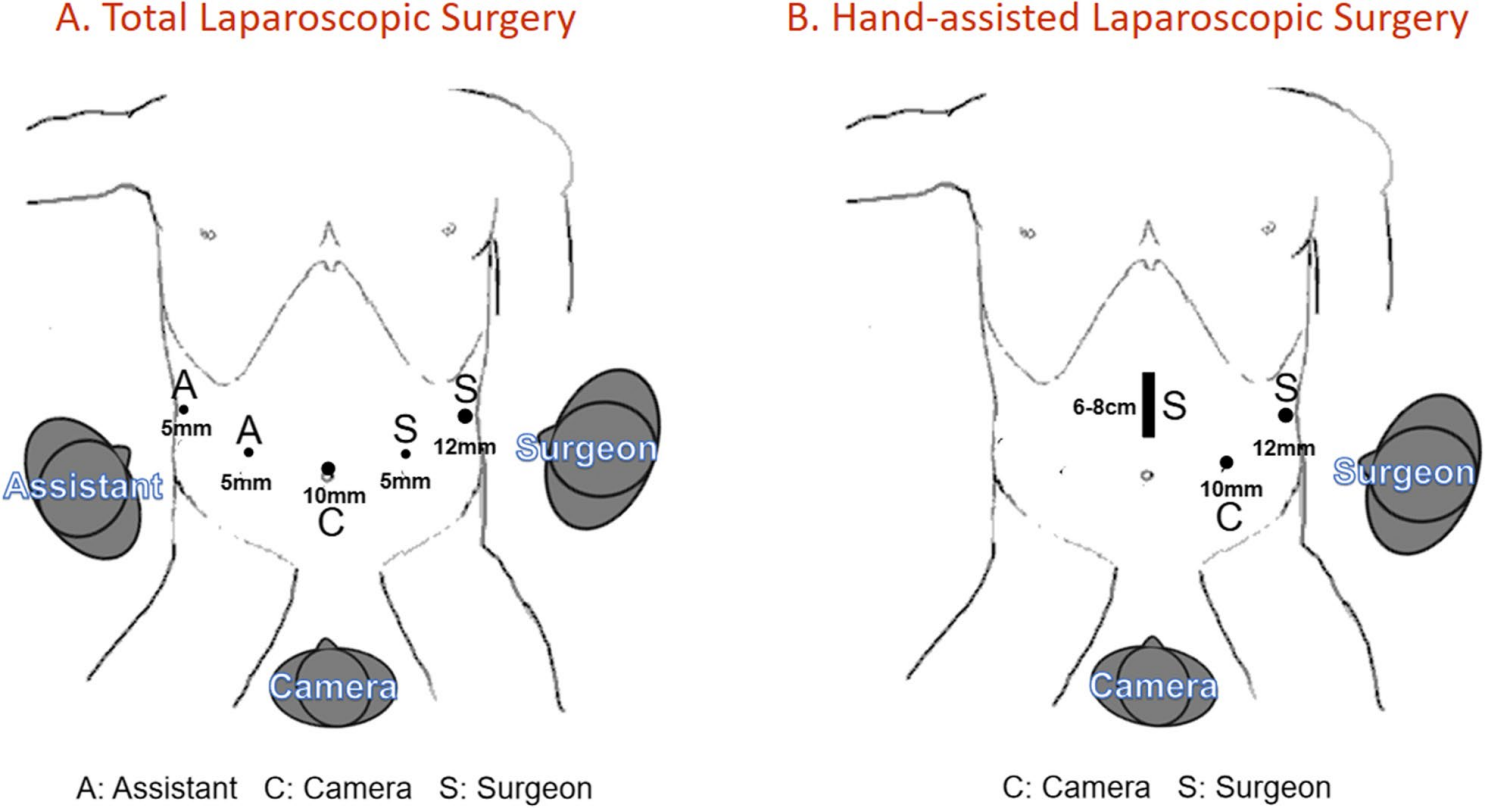
Trocar placement in total laparoscopic surgery (**A**) and hand-assisted laparoscopic surgery (**B**)

The operation proceeded in the following manner: the patient was placed in the reverse Trendelenburg position with the head elevated approximately 15° to 20° and the left side tilted up at approximately 20° to 30°. The surgery was performed with the patient under general and epidural anesthesia in a supine position. The patient was usually placed with the legs separated. The surgical table was reduced by approximately 10–20° into the reverse Trendelenburg position. Therefore, the patient’s upper body was elevated, causing the intestines to gravitate toward the lower abdomen and thus exposing the upper abdomen. The 5-port method was generally used in the TLS (Fig. 2a). A 10-mm trocar was inserted 1 cm below the umbilicus as an observation port. Another 12-mm trocar was introduced in the left anterior axillary line 2 cm below the costal margin as a major hand port. A 5-mm trocar was then inserted into the left midclavicular line 2 cm above the umbilicus as a tractive port. Two 5-mm trocars were placed in the right midclavicular line 2 cm above the umbilicus and in the right anterior axillary line 2 cm below the costal margin as two accessory ports. Generally, the surgeon stands on the patient’s left side, the assistant is on the right side, and the camera operator is between the patient’s legs [5].

The HLS simplified the deployment of surgical staff and used a 2-port method combined with an auxiliary incision (Fig. 2b). The main operating incision was placed in the same way as in the TLS group. The method of inserting the major hand port was the same as that in the TLS group. A 10-mm trocar was inserted in the left midclavicular line 2 cm above the umbilicus as an observation port. A 7-cm skin incision was made in the mid-upper abdomen; then, a lap disc abdominal wall sealing device was inserted through the mini-laparotomy wound to prevent the leakage of carbon dioxide gas. The left hand of the surgeon entered the abdominal cavity through the blue disc, and the right hand used the ultrasonic knife. The pneumoperitoneum was maintained with carbon dioxide gas at a pressure of 12–15 mmHg during surgery. Attention was paid to the distance between the resection margin and the margin of the tumor to ensure R0 resection while maintaining the integrity of the tumor capsule and the negative resection margin. To avoid tumor rupture, excessive force was avoided when clamping the tissue.

In the case of TLS, the tumor specimen was placed in a pre-sewed specimen bag in the abdominal cavity and then removed through a 12 mm trocar or a 5 cm arc incision in the umbilicus based on the actual size of the tumor. In the case of HLS, tumor specimens were bagged and removed through a 7 cm skin incision in the mid-upper abdomen.

**Fig. 3:**
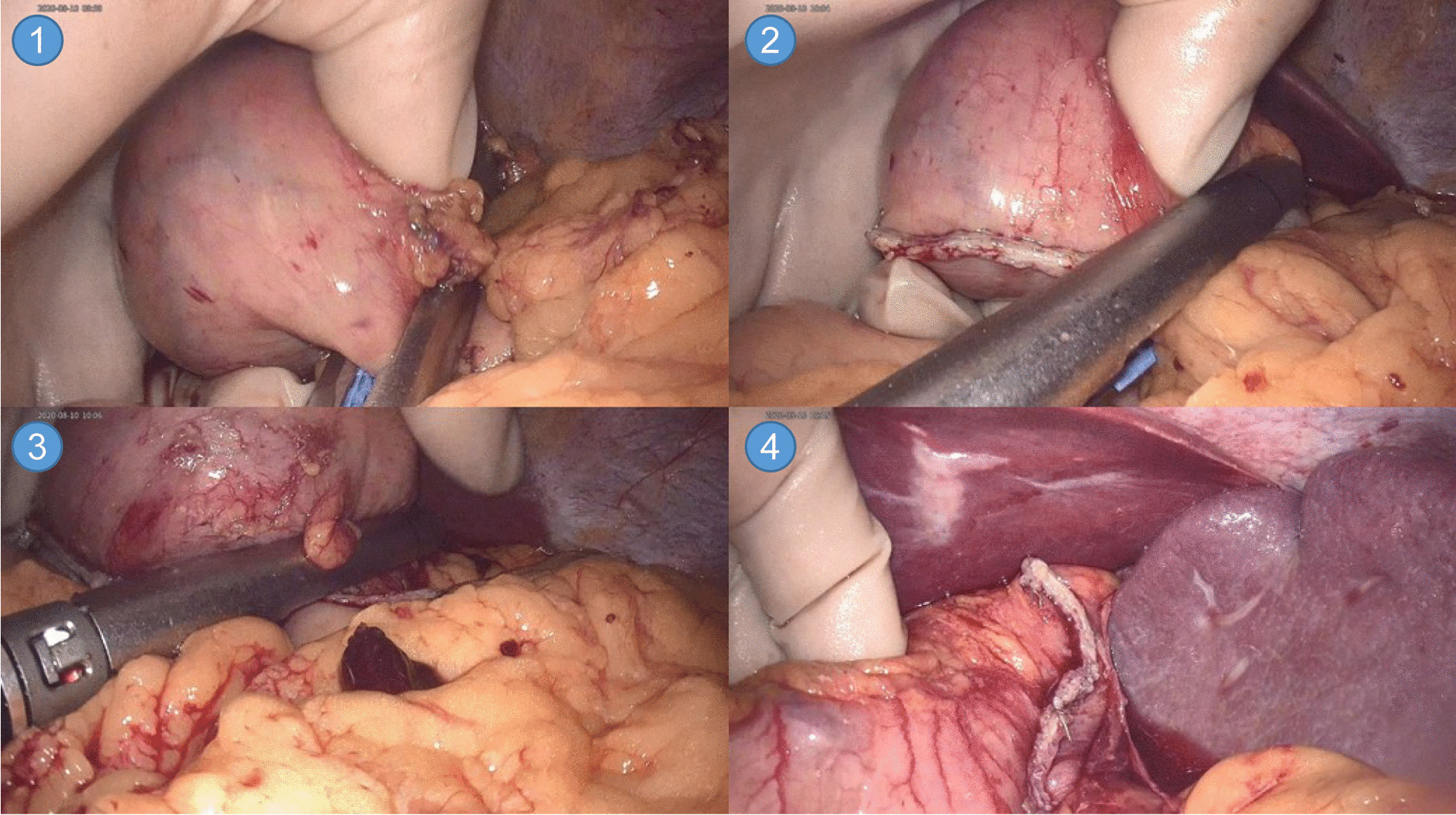
Hand-assisted laparoscopic resection of gastric antrum gastrointestinal stromal tumors

This study was conducted retrospectively. The choice of surgical method was based on both the patient’s condition and the surgeon’s experience. Each patient was given detailed information about the instruments, sizes of the incisions, oncological risks, and the cost of the operation for both the hand-assisted laparoscopic and total laparoscopic approaches. Written informed consent was obtained from all patients before the operation. Tumors in different locations can be resected in the following ways: (1) When a tumor is in the pylorus, the surgeon uses the ultrasonic knife to separate the tissue and uses the left hand to lift the tumor and the stomach wall. According to the tumor size and location, a linear cutting suture device is used to remove the tumor along the periphery at once or in stages. With the advantages of flexible operation and grasping of the left hand, it is possible to preserve normal stomach tissue to avoid pyloric stenosis (Fig. 3). (2) When a tumor is in the lesser curvature of the stomach, the surgeon uses the linear cutting suture device to perform tumor resection after separating the gastrohepatic ligament. (3) When a tumor is in the greater curvature of the stomach, the surgeon can lift the stomach wall and perform tumor resection after freeing the gastrocolic and gastrosplenic ligaments. (4) When a tumor is in the cardia of the stomach, the surgeon first separates the gastrohepatic, gastrosplenic, and gastrophrenic ligaments to fully expose the gastric cardia. The surgeon should try to completely excise the tumor as much as possible, avoiding the esophagus under the protection and precise control of the left hand. All 53 cases were successfully conducted laparoscopically, with none converted to open procedures.

For both the HLSs and TLSs, a plastic bag was introduced into the abdominal cavity to remove the resected specimen. The plastic bag allowed compression of the soft specimen and extraction through a small incision, without contamination of the wound. The specimen in the HLS was delivered out of the abdominal cavity through a 7-cm skin incision. Skin incisions were sutured in a layer-to-layer fashion. Adjuvant imatinib was recommended for patients with a significant risk of recurrence (intermediate or high risk) [8, 9]. In this study, tumor size was defined as the maximum tumor diameter [10]. The mitotic rate was defined as the number of mitoses per 50 high-power fields. Postoperative complications were graded by the Clavien–Dindo classification. The Modified National Institutes of Health criteria were used to grade the risk of the GISTs [10]. All patients were recommended for standard postoperative follow-up every 3–6 months for the first 2 years, every 6–12 months from the 3rd to 5th years, which included a physical examination, chest radiography, laboratory testing, and computed tomography or abdominopelvic ultrasonography. Most routine follow-up appointments. We have supplemented the follow-up results of all patients. Overall survival (OS) represents the time from surgery to the last follow-up or death. All patients were followed up until January 2022.

### Statistical analysis

SPSS statistical software (version 22.0; SPSS Inc.) and R software (version 3.6.1; R Foundation for Statistical Computing) were used for the analysis of all data. The quantitative data by normal distribution were expressed as the mean ± SD, and categorical variables are presented as frequencies and percentages. Differences between the groups were assessed using the t-test, Fisher’s exact test, the Mann–Whitney U test, or the χ2 test. Survival rates were calculated using the Kaplan–Meier method and compared using the log-rank test. All tests were two-sided with a significance level of *P* < 0.05.

## Results

### Clinicopathological characteristics

The clinical and pathologic characteristics of the TLS and HLS groups are provided in Table 1. The mean age was 56.9 years in the TLS group and 58.1 years in the HLS group (*P* = 0.691). The mean tumor sizes in the TLS group and the HLS group were 3.9 ± 1.7 cm and 4.4 ± 2.2 cm, respectively (*P* = 0.317). The numbers of cases of cardia, fundus, body, and antrum gastric GISTs were 5, 8, 9, and 14, respectively, in the TLS group, whereas in the HLS group, the numbers were 7, 3, 6, and 1, respectively. There were no significant differences in sex (*P* = 0.111), BMI (*P* = 0.389), size (*P* = 0.126), American Society of Anesthesiologists physical status (*P* = 0.789), comorbidity (*P* = 0.437), mitotic count (*P* = 0.713), or modified NIH criteria risk (*P* = 0.473) between the two groups.

**Table 1:** Basic characteristics of the total laparoscopic surgery and hand-assisted laparoscopic surgery groups

Characteristic	Mean. (SD) / No. (%)		*P* Value
	TLS group (n = 36)	HLS group (n = 17)	
Age, y	56.9 ± 8.9	58.1 ± 11.4	0.691
BMI, kg/m^2^	22.6 ± 2.7	23.3 ± 2.9	0.389
Size, cm	3.9 ± 1.7	4.4 ± 2.2	0.317
Size, cm			0.126
≤ 5	32 (88.9%)	12 (70.6%)	
> 5	4 (11.1%)	5 (29.4%)	
Sex			0.111
Female	19 (52.8%)	5 (29.4%)	
Male	17 (47.2%)	12 (70.6%)	
ASA-PS			0.789
1	26 (72.2%)	11 (64.7%)	
2	9 (25.0%)	5 (29.4%)	
3	1 (2.8%)	1 (5.9%)	
Comorbidity			0.437
None	27 (75.0%)	11 (64.7%)	
Yes	9 (25.0%)	6 (35.3%)	
Tumor location			0.032
Gastric cardia	5 (13.9%)	7 (41.2%)	
Gastric fundus	8 (22.2%)	3 (17.7%)	
Gastric body	9 (25.0%)	6 (35.3%)	
Gastric antrum	14 (38.9%)	1 (5.9%)	
Mitotic count (per 50 HPFs)			0.713
≤ 5	26 (72.2%)	14 (82.4%)	
6–10	6 (16.7%)	2 (11.8%)	
> 10	4 (11.1%)	1 (5.9%)	
Modified NIH criteria			0.473
Very low risk	4 (11.1%)	2 (11.8%)	
Low risk	22 (61.1%)	8 (47.1%)	
Intermediate risk	6 (16.7%)	6 (35.3%)	
High risk	4 (11.1%)	1 (5.9%)	

*BMI* body mass index; *ASA-PS* American Society of Anesthesiologists physical status, *HPFs* high-power fields, *NIH* National Institutes of Health, *TLS* total laparoscopic surgery, *HLS* hand-assisted laparoscopic surgery, *SD* standard deviation

### Surgical outcomes

The surgical outcomes are presented in Table 2. Compared with the TLS group, the HLS group had less intraoperative blood loss (34.7 mL vs. 58.3 mL, *P* = 0.006), shorter operative time (70.6 min vs. 134.4 min, *P* < 0.001), longer incision length (7.6 cm vs. 6.7 cm, *P* = 0.001), and larger maximum tumor margin (3.0 cm vs. 2.3 cm, *P* = 0.004). Tumor rupture occurred in one patient in the TLS group during surgery.

**Table 2:** Surgical outcomes, postoperative recovery, morbidity, and mortality in the laparoscopic surgery and hand-assisted laparoscopic surgery groups

Outcome	Mean. (SD) / No. (%)		*P* Value
	TLS group (n = 36)	HLS group (n = 17)	
*Surgical outcome*			
Estimated blood loss (mL)	58.3 ± 30.4	34.7 ± 21.0	0.006
Operative time (minutes)	134.4 ± 53.7	70.6 ± 19.1	< 0.001
Length of incision, cm	6.7 ± 1.0	7.6 ± 1.5	0.001
Maximum margin of tumor, cm	3.0 ± 0.8	2.3 ± 0.9	0.004
Tumor rupture during surgery	1 (2.8%)	0 (0.0%)	0.488
Tumor resection margin R0	0 (0.0%)	0 (0.0%)	–
Open conversion	0 (0.0%)	0 (0.0%)	–
*Postoperative recovery*			
Time to first flatus (days)	2.7 ± 0.5	1.8 ± 0.4	< 0.001
Time to ambulation (days)	1.6 ± 0.5	1.2 ± 0.4	0.005
Time to first liquid intake (days)	3.7 ± 0.5	2.9 ± 0.5	< 0.001
Time to first semifluid intake (days)	5.5 ± 0.5	4.4 ± 0.7	< 0.001
Postoperative hospital stays (days)	7.2 ± 2.1	7.9 ± 2.0	0.273
Reoperation	0 (0.0%)	0 (0.0%)	–
Unplanned readmission	0 (0.0%)	0 (0.0%)	–
Morbidity type*			
Postoperative complication*	3 (8.3%)	1 (5.9%)	0.753
Wound problem	1 (2.8%)	1 (5.9%)	0.580
Pulmonary	2 (5.6%)	1 (5.9%)	0.962
Leakage	0 (0.0%)	0 (0.0%)	–
Hemorrhage	1 (2.8%)	0 (0.0%)	0.488
Mortality	0 (0.0%)	0 (0.0%)	–
*Clavien–Dindo classification*^†^			> 0.999
I	0 (0.0%)	0 (0.0%)	
II	2 (5.6%)	1 (5.9%)	
IIIa	1 (2.8%)	0 (0.0%)	
IIIb	0 (0.0%)	0 (0.0%)	
IV	0 (0.0%)	0 (0.0%)	
V	0 (0.0%)	0 (0.0%)	

*TLS* total laparoscopic surgery, *HLS* hand-assisted laparoscopic surgery, *SD* standard deviation

^*^One patient may had more than one morbidity type

^**†**^Clavien-Dindo classification only calculates the highest grade of complications

### Postoperative recovery, complications, and prognosis

**Fig. 4:**
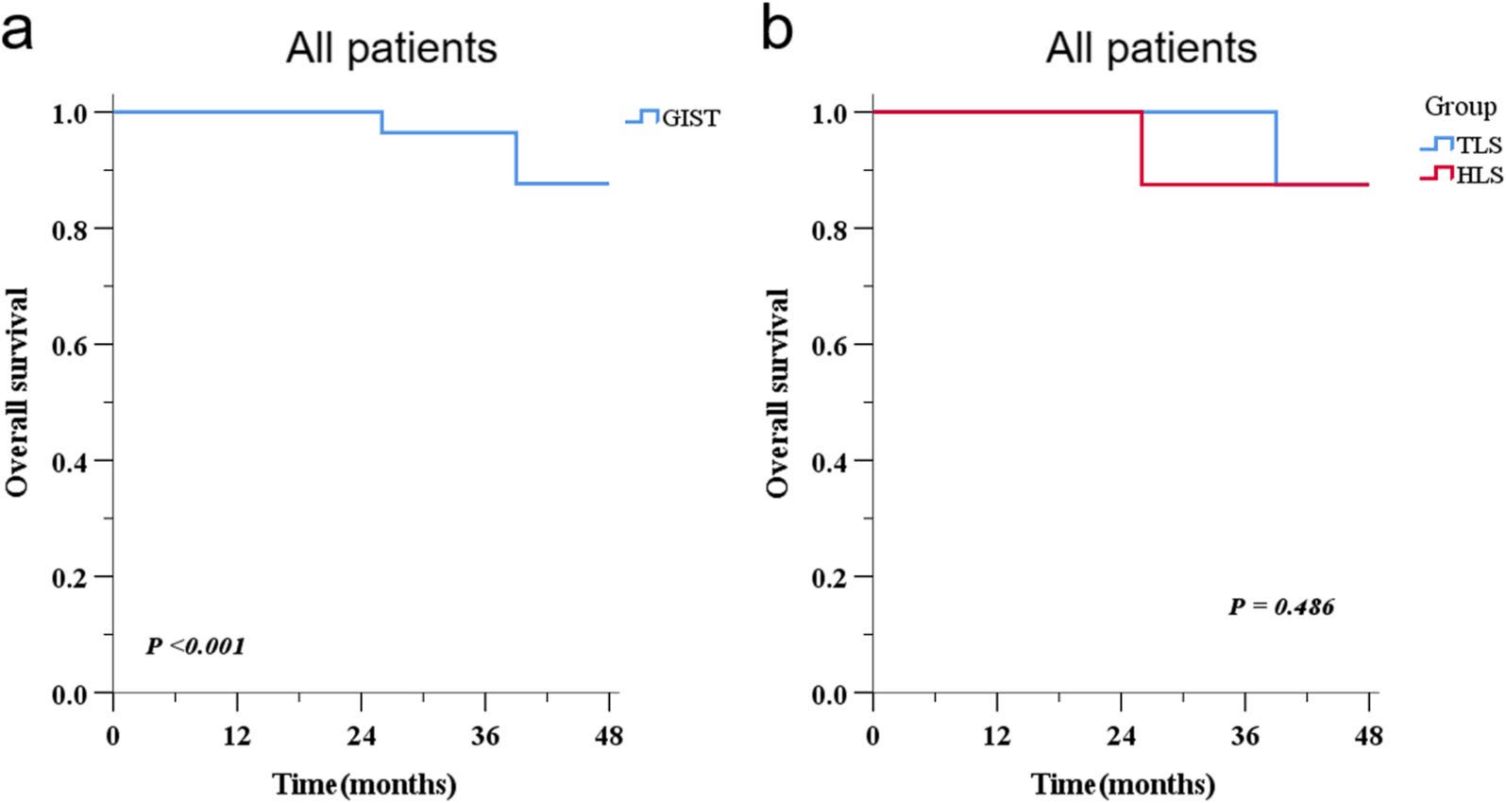
Overall survival. **a** OS of all patients; **b** OS of patients in the TLS and HLS groups

Table 2 showed the postoperative recovery and complications. The recovery course, time to ambulation (1.2 d vs. 1.6 d, *P* = 0.005), time to first flatus (1.8 d vs. 2.7 d, *P* < 0.001), time to first liquid intake (2.9 d vs. 3.7 d, *P* < 0.001), and time to first solid food intake (4.4. d vs. 5.5 d, *P* < 0.001) were all significantly shorter in the HLS group than in the TLS group. There was no significant difference in the postoperative hospital stay between the HLS and TLS groups (7.9. d vs. 7.2 d, *P* = 0.273). There was no reoperation and unplanned readmission in both groups of patients. For the postoperative complication, no significant differences were found between the HLS and TLS groups in terms of incidence (1 of 17 patients [5.9%] vs. 3 of 36 patients [8.3%], respectively; P = 0.753). For specific complications, such as wound problem (1 of 17 patients [5.9%] vs 1 of 36 patients [2.8%]), pulmonary (1 of 17 patients [5.9%] vs 2 of 36 patients [5.6%]), leakage (0 of 17 patients [0%] vs 0 of 36 patients [0%]), and hemorrhage (0 of 17 patients [0%] vs 1 of 36 patients [2.8%]), there was no significant difference between the two groups. For Clavien-Dindo classification (I-V grade), no significant difference was found between the two groups (*P* > 0.05), and there were no postoperative deaths in either the HLS group or the TLS group. Figure 4 shows the OS of all the patients as well as the two groups. We found that the 3-year OS of all patients was 93% (95% CI: 88%-98%), and there was no significant difference in the OS between the TLS and HLS groups (*P* = 0.467).

### Adjuvant treatment and oncologic outcomes

Eleven patients in the TLS group and six patients in the HLS group underwent adjuvant Imatinib therapy (400 mg, QD) for 3 to 24 months. The median follow-up duration for the entire cohort was 26 months (range, 1- 48 months). Four patients had recurrent disease, including two with local recurrence and two with metastatic disease. One patient who was at high risk died of the disease in the TLS group (Table 3).

**Table 3:** Adjuvant treatment and survival outcomes

	TLS group (n = 36)	HLS group (n = 17)	*P* Value
*Adjuvant imatinib treatment (imatinib)*			0.730
No	25	11	
Postoperative	11	6	
*Survival outcomes*			0.753
No recurrence	33	16	
Recurrence	2	1	
Dead	1	0	

*TLS* total laparoscopic surgery, *HLS* hand-assisted laparoscopic surgery

## Discussion

GISTs, the most common soft tissue sarcomas of the GI tract, most commonly contain KIT- or PDGFRA-activating mutations and are derived from mesenchymal neoplasms in the GI tract [11–13]. The main clinical symptoms are abdominal pain, bloating, and bleeding in the digestive tract. The prognosis is generally good, with a five-year survival rate of over 70% [14]. The main prognostic tools include biopsy, risk stratification, and whether the tumor ruptured during surgery. Complete surgical resection is regarded as the main treatment method for gastric GISTs. Because lymph nodes are not the main location of metastasis, complete resection of the tumor is emphasized. In 1992, Lukaszczry et *al.* first reported the successful laparoscopic resection of a gastric GIST [15]. In recent years, due to the rapid development of laparoscopic technology, laparoscopic resection has become the main surgical method for the treatment of gastric GISTs [2–4]. However, the space information of the tumor size and scope during surgery is portrayed by the secondary conversion function of laparoscopy, which may affect the surgeon’s accurate assessments. Precise positioning during surgery is particularly important for gastric GISTs requiring local resection. HLS can not only avoid the large trauma caused by open surgery but can allow for manual manipulation of the tumor to achieve accurate resection during the operation. HLS can be used for the surgical treatment of gastric GISTs to maximize the preservation of normal gastric tissue and gastric function while avoiding esophageal or pyloric stenosis [5].

Our results confirmed that the operation time of HLS was significantly shorter than that of TLS. Under the same type of operation, the trauma due to HLS was less, resulting in a shorter time to ambulation, a shorter time to first flatus, and a shorter time to first diet compared to TLS. The possible reasons for this include: first, with HLS, the surgeon can directly palpate the tumor, bringing about a more accurate position; and second, HLS can better expose the relevant anatomical structures around the tumor to facilitate tissue separation and tumor resection. In addition, HLS has lower requirements for the surgeon's laparoscopic technology, while TLS has higher requirements and a certain learning curve [6, 16–20]. Consequently, the operation time of HLS was significantly less than that of TLS in our study. For HLS, the maximum margin of the tumor was significantly less than that of TLS, ensuring R0 resection. This is because the stomach wall has a certain ability to contract, so the hand can control the range of tumor resection more precisely. In addition, due to the assistance of the hand, the HLS is more convenient and stable when using the Endo-GIA for surgical stapling. At present, there is still controversy about the laparoscopic resection of gastric GISTs, especially for large tumors and tumors located in the cardia and pylorus. This is because prospective studies are lacking as this type of surgery is difficult to perform, and it sometimes requires a total gastrectomy, a distal gastrectomy, or even a combined organ resection.

Therefore, the guidelines recommend that laparoscopic surgery is mainly used for tumors less than 5 cm and located in the greater curvature of the stomach or in an easy-to-remove location in the lesser curvature [1, 21]. For such patients, there were obvious advantages to HLS. The effect of hand-assisted surgery makes the operation similar to open surgery, where fatal bleeding during the operation can be better controlled [20–22]. Additionally, the surgeon can complete the tumor resection, reduce the probability of conversion to open surgery and preserve normal stomach tissue and gastric function. Simultaneously, HLS can avoid the esophageal or pyloric stenosis caused by the local resection of tumors in specific areas. Furthermore, there was no statistically significant difference between the two groups in terms of intraoperative bleeding or postoperative complications such as gastric bleeding, postoperative wound infections, or wound leakage. This proved that the effects of HLS were equivalent to those of TLS. However, the operation time was significantly shortened in the HLS, and much of the normal gastric tissue was preserved as much as possible. In the treatment of GISTs located in the cardia or pyloric, HLS can decrease the probability of cardia and pyloric stenosis or the possibility of an expanded surgery.

HLS can better allow surgeons who have experienced open surgery to make sense of the points of laparoscopic technology, which serves as a bridge between open surgery and TLS. In terms of the smooth learning curve of HLS, it is easier for most surgeons to accept [6]. Furthermore, by analyzing nearly 20 cases, we found that HLS has unique advantages in the treatment of gastric GISTs, especially for tumors located in the cardia, pylorus, and other difficult areas. HLS has a significantly shorter operation time than TLS, which indicates that patients may have a better postoperative recovery. Moreover, it is easier for GI surgeons, who start laparoscopy in the initial stage, to learn and master the laparoscopic technology of a GIST resection, which helps improve the self-confidence of the surgeon.

Some researchers have also attempted to introduce robotic platforms in the field of GIST surgical resection. High-definition and three-dimensional magnified imaging and a simulated wrist with seven degrees of freedom have greatly improved the flexibility of operation, making conventional laparoscopy, a difficult operation, simple and convenient. However, robotic surgery is still limited by cost, making it difficult to popularize robotic surgery widely [23, 24].

We believe that TLS remains a globally recognized surgical method for GIST. It has the advantages of being a minimally invasive surgery, with smaller surgical incisions and quicker postoperative recovery. However, laparoscopic surgery lacks three-dimensional vision and tactile feedback, which limits its application in complex surgical operations. While the HLS technology can result in direct contact with the internal organs of the abdominal cavity, restore the touch of the hand, identify the tissue, control the bleeding, and help increase the exposure, making up for the disadvantages of the TLS [24–26]: (1) restores the tactile sensation of the hand, improves hand–eye coordination, and at the same time greatly reduces the risk of rupture during GIST; (2) the best surgical field can be obtained by assisted hand pulling, which makes cutting, suturing, and knotting easier, and significantly reduces the difficulty of surgery; (3) the use of auxiliary hands and energy devices can result in better handling of blood vessels well, control accidental bleeding, and improve the safety of surgery; (4) the excised specimen can be removed through the hand-assisted incision, and due to the protection of the hand-assisted device, incision implantation caused by specimen removal can be effectively avoided. All of these can significantly reduce the time and risk during the operation, and our study also confirmed that HLS leads to less blood loss and less operative time, HLS is especially suitable for beginners who are transitioning from open surgery to fully laparoscopic surgery.

Although this study summarizes the advantages of HLS, it still has the following limitations. First, although the data in this study came from a high-volume institution’s database, the sample size was still small, which makes it impossible to perform statistical methods such as propensity score matching to balance the baseline data of patients, such as whether there was a significant difference in the tumor location. Second, long-term survival analysis was lacking. Finally, although HLS can be an optional transitional stage of TLS for novices, TLS is still the main method of gastric GIST surgery. Prospective randomized studies using multicenter data may be needed before a definite answer could be given concerning the surgical value of HLS compared with TLS.

In conclusion, our results confirmed that HLS in gastric stromal tumor resection has advantages, including a shorter operation time, minimal invasiveness, and maximum preservation of gastric function, especially for patients with gastric cardia GISTs. It is an effective surgical method for GI surgeons who have not fully mastered laparoscopic techniques.

## Data Availability

The datasets generated during and analyzed during the current study are not publicly available due to privacy and ethical restrictions but are available from the corresponding author on reasonable request.

## Abbreviations

TL: Total laparoscopic surgery
HLS: Hand-assisted laparoscopic surgery
SD: Standard deviation
BMI: Body mass index
ASA-PS: American Society of Anesthesiologists physical status
HPFs: High-power fields
NIH: National Institutes of Health
